# Progress in Neurology

**Published:** 1902-08-30

**Authors:** 


### Progress in Neurology.

**Death in Epilepsy.**—Spratling,[1] from a large experience at the Craig Colony, says that any epileptic may die suddenly at any time. It may occur suddenly in any case as the result of a simple seizure, such a result, however, is not very common. It may result from a series of fits occurring in rapid succession, and ending in status epilepticus. This causes the death of about one epileptic out of every four who die. Status develops wholly irrespective of the type of epilepsy from which the individual suffers, whether it be grand mal, petit mal, psychic, or Jacksonian. It should be feared when more than three or four well-marked and distinct attacks occur within an hour's time. About one-half as many epileptics die from accidents during a fit as from status epilepticus. The period of greatest danger is during sleep, from the patient rolling over and burying his face in the bedclothes. Epileptics should, therefore, sleep on a hard, flat, hair pillow, or, better still, on none at all, and as far as possible they should be under some supervision while they are asleep.

**Paralyses in Whooping-cough.**—Valentine[2] has recently collected from medical literature and analysed a number of paralyses occurring in whooping-cough. Neuroses and psychoses are rare, the commoner affections being convulsions or paralyses; these are met with mostly between the ages of two and six years. The cerebral paralyses were generally monoplegia or hemiplegia, and, very rarely, paraplegia. Right-sided hemiplegia is often accompanied by aphasia. Paralyses from bulbar or spinal lesions are distinctly rare. The nervous lesions underlying the paralyses are hæmorrhage and softening. The prognosis is grave; many die, many remain permanently paralysed and enfeebled, whilst only two-fifths recover completely.

**Relation of Mental Disorders to Bodily Disease.** Nathan Raw,[3] in an excellent paper, describes the various mental conditions dependent on bodily disease. In heart disease, especially towards the close, mental symptoms may occur, of very variable character, delusions of suspicion and persecution, for instance; rarely they are suicidal, they are especially irritable, and, exceptionally, maniacal. There is no special form of insanity associated with kidney disease, but in the later stages of chronic renal disease the patients may exhibit marked delusions of fear and suspicion, and they are often delirious, restless, irritable, and show mental failure, until coma terminates the scene. Patients have been sent to an asylum as lunatics when in this condition. In exophthalmic goitre the first symptom, in many cases, is a sudden change in the mental condition; the patient becomes irritable, excitable, and flighty; her moral nature, too, is altered, and she is untruthful, spiteful, and suspicious. Delusions of impending danger are developed, and she has all the appearances of a melancholic. In myxœdema, though in general there is a slowness of perception and conduction, acute attacks of insanity, either mania or melancholia, may develop. In diabetes, the patient is often irritable, fretful, and impatient in the earlier stages, and in the later ones he may become suspicious and distrustful, and may even develop delusions. In typhoid fever there are two distinct phases of mental disorder—one in the acute febrile stage, and the other in convalescence. In the first stage, the patient may rapidly pass into a condition of raving mania, most difficult to treat or control, or into one of wakefulness, with hallucinations of vision and hearing and even delusions which may terminate in a sleep unto death. During the third week there may be profound mental prostration or actual imbecility. Post-typhoid insanity may occur, but the patient practically always makes a good recovery. In the early stages of acute pneumonia, wild delirium and even mania may be marked, and such cases are very fatal. The sleeplessness is due to cerebral irritation at first and subsequently to exhaustion. Insanity never follows pneumonia. Acute mania and delusional insanity followed by profound melancholia may occur as a result of removal of the testes for diseases of the prostate. The same dire effects may follow operations on the healthy ovaries. We should therefore hesitate before removing these organs when in a healthy state for any disease whatever. Acute mania may follow the application of atropine to the eye in elderly people; the prognosis is fortunately good. Raw is convinced that a large number of people are certified as lunatics who are simply temporarily insane owing to some bodily disorder or toxic poisoning. Such people should not have the stigma of being detained in an asylum, but should be treated in a hospital for mental diseases.

**Abiotrophy** is a term introduced by Gowers[4] to indicate decay or degeneration in consequence of a defect of vital endurance. Early baldness is an illustration. In the various forms of idiopathic muscular atrophy, in which there is a primary atrophy of the muscular fibres we have examples of a true abiotrophy. The term includes both simple muscular atrophy and pseudo-hypertrophic paralysis. The great variation in the interstitial process compels us to regard the failure of life in the muscular fibres as the essential element in the disease. The connective tissues may or may not maintain the normal bulk of the muscles. The essential unity of these diseases is further shown in the early wasting, in the pseudohypertrophic form, of the lower part of the pectoralis major and the latissimus. In such primary muscular atrophies the distribution of the affection is strikingly different from that of those atrophies which depend on the nervous system—the muscles of the extremities, the hands and feet, suffer least. Abiotic degeneration may occur in the nervous system. Decay occurs first in those parts of the axis cylinder processes which are most remote from their cells of origin. The death of the nerve fibres, if in the central nervous system, is accompanied by an increase of the interstitial tissue—the neuroglia—so that for a long time the nature of the process was misunderstood. The spinal motor neurons may be affected, as in abiotic infantile atrophy, which commences about the end of the first year of life and causes almost universal paralysis and death at the end of the fifth or sixth year. The affection begins in the muscles of the hip and thigh and trunk, and thence extends, reaching last the extremities of the limbs—in this respect resembling abiotic muscular atrophy and differing from the ordinary form of spinal atrophy. An abiotic degeneration of the lateral pyramidal tracts, causing spastic paraplegia, is also met with in early adult life. The optic nerves frequently suffer from abiotic wasting. Many groups of cases are on record where several members of the same family have become blind, usually between the ages of 15 and 25 years, from a slow wasting of the nerve fibres, progressive in spite of treatment. Friedreich's disease is a well-known form of abiotic atrophy, as is also the form sometimes known as heredo-cerebellar ataxy, but which is connected by many links to Friedreich's disease. In all these cases of abiotrophic affections of the nervous system in childhood or early adult life, the affection is generally a family one, and often hereditary. This "family" feature is clear proof of the congenital nature of the tendency to vital failure. The second group of degenerations due to premature vital failure occurs at the other end of life. The neurons which thus decay are the spinal motor neurons—causing either progressive muscular atrophy or labio-glossal paralysis. Mental change, especially simple mental failure, also often occurs under the same conditions, no doubt from a slow degeneration of cerebral neurons which connect and combine others. Another senile malady, paralysis agitans, must also be referred to vital failure in some cerebral motor structure. The commonest degenerations of middle life are tabes and general paralysis, but they are generally the result of some definite cause, and it is doubtful whether the underlying neuronic degenerations can be termed "abiotrophic."

[1] Med. News. [2] Lancet, Feb. 8. [3] Lancet, June 14. [4] Lancet, April 12.

